# Information Measure in Terms of the Hazard Function and Its Estimate

**DOI:** 10.3390/e23030298

**Published:** 2021-02-28

**Authors:** Sangun Park

**Affiliations:** 1 Department of Statistics and Data Science, Yonsei University, Shinchon Dong 134, Seoul 03722, Korea; sangun@yonsei.ac.kr; 2 Department of Applied Statistics, Yonsei University, Shinchon Dong 134, Seoul 03722, Korea

**Keywords:** censoring variable, entropy, fisher information, Kullback-Leibler information, order statistics

## Abstract

It is well-known that some information measures, including Fisher information and entropy, can be represented in terms of the hazard function. In this paper, we provide the representations of more information measures, including quantal Fisher information and quantal Kullback-leibler information, in terms of the hazard function and reverse hazard function. We provide some estimators of the quantal KL information, which include the Anderson-Darling test statistic, and compare their performances.

## 1. Introduction

Suppose that *X* is a random variable with a continuous probability density function (p.d.f.) f(x;θ), where θ is a real-valued scalar parameter. It is well-known that the Fisher information plays an important role in statistical estimation and inference, which is defined as

$$\begin{array}{c} I\left(\theta \right)=\int_{-\infty }^{\infty }{\left\{\frac{\partial }{\partial \theta }\log f\left(x;\theta \right)\right\}}^{2}f\left(x;\theta \right)dx. \end{array}$$

Fisher information identity in terms of the hazard function has been provided by Efron and Johnstone [1] as

(1) $$\begin{array}{c} I\left(\theta \right)=\int_{-\infty }^{\infty }{\left\{\frac{\partial }{\partial \theta }\log h\left(x;\theta \right)\right\}}^{2}f\left(x;\theta \right)dx, \end{array}$$

where h(x;θ) is the hazard function defined as f(x;θ)/(1−F(x;θ)) and F(x;θ) is the cumulative distribution function.

It is also well-known that the entropy (Teitler et al., 1986) and Kullback-Leibler information [2] can be represented in terms of the hazard function, respectively, as

$$\begin{array}{c} H\left(X\right)=1-\int_{-\infty }^{\infty }f\left(x\right)\log h\left(x\right)dx \end{array}$$

and

$$\begin{array}{c} KL\left(f:g\right)=\int_{-\infty }^{\infty }f\left(x\right)\left(\frac{h_{g}\left(x\right)}{h_{f}\left(x\right)}-\log\frac{h_{g}\left(x\right)}{h_{f}\left(x\right)}-1\right)dx, \end{array}$$

where $h_{f}\left(x\right)$ and $h_{g}\left(x\right)$ are the hazard functions defined as f(x)/(1−F(x)) and g(x)/(1−G(x)), respectively.

The *quantal (randomly censored) Fisher information* and the *quantal (randomly censored) Kullback-Leibler information* have been defined [3], respectively, as

$$\begin{array}{c} I_{QF}\left(\theta \right)=\int_{-\infty }^{\infty }\left\{F\left(t;\theta \right){\left(\frac{\partial }{\partial \theta }\log F\left(t;\theta \right)\right)}^{2}+\bar{F}\left(t;\theta \right){\left(\frac{\partial }{\partial \theta }\log\bar{F}\left(t;\theta \right)\right)}^{2}\right\}dW\left(t\right) \end{array}$$

and

(2) $$\begin{array}{c} QKL\left(F:G\right)=\int_{-\infty }^{\infty }\left(F\left(t\right)\log\frac{F(t)}{G(t)}+\bar{F}\left(t\right)\log\frac{\bar{F}\left(t\right)}{\bar{G}\left(t\right)}\right)dW\left(t\right), \end{array}$$

where W(t) is an appropriate weight function which satisfies $\int_{-\infty }^{\infty }dW\left(x\right)=1$.

The quantal Fisher information is related with the Fisher information in the ranked set sample, and the quantal Kullback-Leibler information is related with the cumulative residual entropy [4] and cumulative entropy [5], defined as

(3) $$\begin{array}{c} CRE\left(F\right)=-\int_{0}^{\infty }\left(1-F\left(x\right)\right)\log\left(1-F\left(x\right)\right)dx \end{array}$$

and

(4) $$\begin{array}{c} CE\left(F\right)=-\int_{0}^{\infty }F\left(x\right)\log F\left(x\right)dx. \end{array}$$

The information representation in terms of the cumulative functions enables us to estimate the information measure by employing the empirical distribution function.

The organization of this article is as follows: In Section 2, we discuss the relation between the quantal Fisher information and quantal Kullback-Leibler information. In Section 3, we provide the expression of the quantal Fisher information in terms of the hazard and reverse hazard functions as

$$\begin{array}{c} I_{QF}\left(\theta \right)=\int_{-\infty }^{\infty }W\left(x\right){\left(\frac{\partial }{\partial \theta }\log r\left(x;\theta \right)\right)}^{2}f\left(x;\theta \right)dx-\int_{-\infty }^{\infty }W\left(x\right){\left(\frac{\partial }{\partial \theta }\log h\left(x;\theta \right)\right)}^{2}f\left(x;\theta \right)dx, \end{array}$$

where h(x;θ) and r(x;θ) are the hazard and reverse hazard functions, respectively.

We also provide the expression of the *quantal (randomly censored)* KL information in terms of the hazard and reverse hazard functions as

$$\begin{array}{c} QKL\left(F:G\right)=\int_{-\infty }^{\infty }W\left(x\right)f\left(x\right)\left\{\frac{r_{g}\left(x\right)}{r_{f}\left(x\right)}-\log\frac{r_{g}\left(x\right)}{r_{f}\left(x\right)}-\frac{h_{g}\left(x\right)}{h_{f}\left(x\right)}+\log\frac{h_{g}\left(x\right)}{h_{f}\left(x\right)}\right\}dx, \end{array}$$

where $r_{f}\left(x\right)$ and $r_{g}\left(x\right)$ are the reverse hazard functions defined as f(x)/F(x) and g(x)/G(x), respectively.

This representation enables us to estimate the quantal information by employing the nonparametric hazard function estimator. In Section 4, we discuss the choice of the weight function W(x) in terms of maximizing the related Fisher information. In Section 5, we provide the estimator of (2) and evaluate its performance as a goodness-of-fit test statistic. Finally, in Section 6, some concluding remarks are provided.

## 2. Quantal Fisher Information and Quantal Kullback-Leibler Information

If we define the quantal response variable *Y* at *t* as

$$\begin{array}{c} Y=\left\{\begin{array}{cc} 1\;if & X\leq \;t\\ 0\;if & X>t, \end{array}\right. \end{array}$$

its density function is

$$\begin{array}{c} f_{Y}\left(y:\theta \right)=F{\left(t;\theta \right)}^{y}\bar{F}{\left(t;\theta \right)}^{1-y}. \end{array}$$

Then, the conditional Fisher information in the quantal response at *t* about θ can be obtained as

$$\begin{array}{c} I_{QF}^{t}\left(\theta \right)=F\left(t;\theta \right){\left(\frac{\partial }{\partial \theta }\log F\left(t;\theta \right)\right)}^{2}+\bar{F}\left(t;\theta \right){\left(\frac{\partial }{\partial \theta }\log\bar{F}\left(t;\theta \right)\right)}^{2}. \end{array}$$

This conditional Fisher information has been studied in terms of censoring by Gertsbakh [6] and Park [7], and its weighted average has been defined to be the *quantal randomly censored Fisher information* [3] as

(5) $$\begin{array}{c} I_{QF}\left(\theta \right)=\int_{-\infty }^{\infty }\left\{F\left(t;\theta \right){\left(\frac{\partial }{\partial \theta }\log F\left(t;\theta \right)\right)}^{2}+\bar{F}\left(t;\theta \right){\left(\frac{\partial }{\partial \theta }\log\bar{F}\left(t;\theta \right)\right)}^{2}\right\}dW\left(t\right), \end{array}$$

where W(t) is an appropriate weight function. The expression (5) says that $I_{QF}\left(\theta \right)$ may be called *cumulative Fisher information* and can be written in a simpler way, as

$$\begin{array}{c} I_{QF}\left(\theta \right)=\int_{-\infty }^{\infty }\frac{{\left(\frac{\partial }{\partial \theta }F\left(x;\theta \right)\right)}^{2}}{F(x;\theta )(1-F(x;\theta ))}dW\left(x\right). \end{array}$$

**Remark** **1.** *If we take W(x) to be F(x;θ), $I_{QF}\left(\theta \right)$ is related with the Fisher information in the ranked set sample [8] as*

$$\begin{array}{c} I_{RSS}\left(\theta \right)=I_{SRS}\left(\theta \right)+n\left(n+1\right)I_{QF}\left(\theta \right), \end{array}$$

*where $I_{SRS}\left(\theta \right)$ is the Fisher information in a simple random sample of size n, which is equal to nI(θ), and $I_{RSS}\left(\theta \right)$ is the Fisher information in a ranked set sample.*

*The result means that the ranked set sample has additional ordering information in the n(n+1) pairs to the simple random sample. Hence, $1+\left(n+1\right)I_{QF}/I\left(\theta \right)$ represents the efficiency level of the ranked set sample relative to the simple random sample.*

In a similar way, the Kullback-Leibler (KL) information between two quantal random variables can be obtained as

$$\begin{array}{c} KL_{t}\left(F:G\right)=F\left(t\right)\log\frac{F(t)}{G(t)}+\bar{F}\left(t\right)\log\frac{\bar{F}\left(t\right)}{\bar{G}\left(t\right)}. \end{array}$$

Then, the weighted average of $KL_{t}\left(F:G\right)$ has been defined to be *quantal (randomly censored) divergence* [3], as

$$\begin{array}{c} QKL\left(F:G\right)=\int_{-\infty }^{\infty }\left(F\left(x\right)\log\frac{F(x)}{G(x)}+\bar{F}\left(x\right)\log\frac{\bar{F}\left(x\right)}{\bar{G}\left(x\right)}\right)dW\left(x\right). \end{array}$$

We note that the quantal KL information (quantal divergence) with dW(x)=dx is equal to the addition of the cumulative KL information (Park, 2015) and cumulative residual KL information [9]. This quantal Kullback-Leibler information has been discussed in constructing goodness-of-fit test statistics by Zhang [10].

The following approximation of the KL information in terms of the Fisher information is well-known [11], as

(6) $$\begin{array}{ccc} KL(f(x;\theta ):f(x;\theta +\Delta \theta )) & \approx & \frac{1}{2}{\left(\Delta \theta \right)}^{2}\int_{-\infty }^{\infty }{\left\{\frac{\partial }{\partial \theta }\log f\left(x;\theta \right)\right\}}^{2}f\left(x;\theta \right)dx. \end{array}$$

Hence, we can also apply Taylor’s expansion to (2) to have the approximation of the quantal KL information in terms of the quantal Fisher information as follows:

**Lemma** **1.** $$\begin{array}{c} QKL\left(F\left(x;\theta \right):F\left(x;\theta +\Delta \theta \right)\right)\approx \frac{1}{2}{\left(\Delta \theta \right)}^{2}I_{QF}\left(\theta \right). \end{array}$$

**Proof** **of** **Lemma 1.** By applying the Taylor expansion, we have

$$\begin{array}{c} QKL(F(x;\theta ):F(x;\theta +\Delta \theta ))\approx \\ \;\;\;\;\;\;\;-\frac{1}{2}{\left(\Delta \theta \right)}^{2}\int_{-\infty }^{\infty }\left\{F\left(x;\theta \right)\frac{\partial^{2}}{\partial \theta^{2}}\log F\left(x;\theta \right)+\bar{F}\left(x;\theta \right)\frac{\partial^{2}}{\partial \theta^{2}}\log\bar{F}\left(x;\theta \right)\right\}dW\left(x\right). \end{array}$$

Then, we can show that

$$\begin{array}{c} -F\left(x;\theta \right)\frac{\partial^{2}}{\partial \theta^{2}}\log F\left(x;\theta \right)-\bar{F}\left(x;\theta \right)\frac{\partial^{2}}{\partial \theta^{2}}\log\bar{F}\left(x;\theta \right)=\\ \;\;\;\;\;\;\;\;\;\;\;\;\;\;\;\;\;\;\;F\left(x;\theta \right){\left(\frac{\partial }{\partial \theta }\log F\left(x;\theta \right)\right)}^{2}+\bar{F}\left(x;\theta \right){\left(\frac{\partial }{\partial \theta }\log\bar{F}\left(x;\theta \right)\right)}^{2}. \end{array}$$

□

## 3. Quantal Fisher Information in Terms of the (Reversed) Hazard Function

It is well-known that the Fisher information can be represented in terms of the hazard function [1] as

(7) $$\begin{array}{c} I\left(\theta \right)=\int_{-\infty }^{\infty }{\left\{\frac{\partial }{\partial \theta }\log h\left(x;\theta \right)\right\}}^{2}f\left(x;\theta \right)dx, \end{array}$$

where h(x;θ) is the hazard function defined as f(x;θ)/(1−F(x;θ)).

The mirror image of (1) provides another representation of the Fisher information in terms of the reverse hazard function [12] as

(8) $$\begin{array}{c} I\left(\theta \right)=\int_{-\infty }^{\infty }{\left\{\frac{\partial }{\partial \theta }\log r\left(x;\theta \right)\right\}}^{2}f\left(x;\theta \right)dx, \end{array}$$

where r(x;θ) is the reverse hazard function defined as f(x;θ)/F(x;θ).

Then, (6) can be written again in terms of both hazard function and reversed hazard function in view of (7) and (8) as follows:

**Lemma** **2.** $$\begin{array}{ccc} KL(f(x;\theta ):f(x;\theta +\Delta \theta )) & = & \frac{1}{2}{\left(\Delta \theta \right)}^{2}\int_{-\infty }^{\infty }{\left\{\frac{\partial }{\partial \theta }\log h\left(x;\theta \right)\right\}}^{2}f\left(x;\theta \right)dx\\ & = & \frac{1}{2}{\left(\Delta \theta \right)}^{2}\int_{-\infty }^{\infty }{\left\{\frac{\partial }{\partial \theta }\log r\left(x;\theta \right)\right\}}^{2}f\left(x;\theta \right)dx. \end{array}$$

Now, we show that the quantal Fisher information can also be expressed in terms of both hazard function and reversed hazard function, as follows.

**Theorem** **1.** *Suppose that W(x) is bounded and the regularity conditions for the existence of the Fisher information hold.*

(9) $$\begin{array}{c} I_{QF}\left(\theta \right)=\int_{-\infty }^{\infty }W\left(x\right){\left(\frac{\partial }{\partial \theta }\log r\left(x;\theta \right)\right)}^{2}f\left(x;\theta \right)dx-\int_{-\infty }^{\infty }W\left(x\right){\left(\frac{\partial }{\partial \theta }\log h\left(x;\theta \right)\right)}^{2}f\left(x;\theta \right)dx. \end{array}$$

**Proof** **of** **Theorem 1.** In view of Park [7], we have the decomposition of the Fisher information as

(10) $$\begin{array}{c} I\left(\theta \right)=I_{L}^{t}\left(\theta \right)+I_{QF}^{t}\left(\theta \right)+I_{R}^{t}\left(\theta \right), \end{array}$$

where

$$\begin{array}{c} I_{L}^{t}\left(\theta \right)=\int_{-\infty }^{t}{\left(\frac{\partial }{\partial \theta }\log r\left(x;\theta \right)\right)}^{2}f\left(x;\theta \right)dx \end{array}$$

and

$$\begin{array}{c} I_{R}^{t}\left(\theta \right)=\int_{t}^{\infty }{\left(\frac{\partial }{\partial \theta }\log h\left(x;\theta \right)\right)}^{2}f\left(x;\theta \right)dx. \end{array}$$

Hence, $I_{QF}^{t}\left(\theta \right)$ can also be expressed from (10) as

(11) $$\begin{array}{c} I_{QF}^{t}\left(\theta \right)=\int_{-\infty }^{t}{\left(\frac{\partial }{\partial \theta }\log h\left(x;\theta \right)\right)}^{2}f\left(x;\theta \right)dx-\int_{-\infty }^{t}{\left(\frac{\partial }{\partial \theta }\log r\left(x;\theta \right)\right)}^{2}f\left(x;\theta \right)dx. \end{array}$$

We can take the expectation of (11) and apply Fubini’s theorem to get the result. □

**Example** **1.** *If W(x) is taken to be F(x;θ), (9) can be written as*

$$\begin{array}{c} I_{QF}\left(\theta \right)=\frac{1}{2}\left(I_{1:2}\left(\theta \right)+I_{2:2}\left(\theta \right)-2I\left(\theta \right)\right) \end{array}$$

*because it has been shown in Park (1996) that*

$$\begin{array}{ccc} I_{1:2}\left(\theta \right) & = & 2\int_{-\infty }^{\infty }{\left(\frac{\partial }{\partial \theta }\log h\left(x;\theta \right)\right)}^{2}f\left(x;\theta \right)\left(1-F\left(x;\theta \right)\right)dx\\ I_{2:2}\left(\theta \right) & = & 2\int_{-\infty }^{\infty }{\left(\frac{\partial }{\partial \theta }\log r\left(x;\theta \right)\right)}^{2}f\left(x;\theta \right)F\left(x;\theta \right)dx, \end{array}$$

*where $I_{i:n}\left(\theta \right)$ is the Fisher information in the ith order statistic from an independently and identically distributed sample of size n.*

## 4. Quantal KL Information and Choice of the Weight Function in Terms of Maximizing the Quantal Fisher Information

Because Lemma 2 shows that the approximation of the Kullback-Leibler information can be represented in terms of the hazard function and reverse hazard function, the following representations of the KL information in terms of the hazard function and reverse hazard function have been shown in Park and Shin [2] as

(12) $$\begin{array}{c} KL\left(f:g\right)=\int_{-\infty }^{\infty }f\left(x\right)\left(\frac{h_{g}\left(x\right)}{h_{f}\left(x\right)}-\log\frac{h_{g}\left(x\right)}{h_{f}\left(x\right)}-1\right)dx \end{array}$$

and

$$\begin{array}{c} KL\left(f:g\right)=\int_{-\infty }^{\infty }f\left(x\right)\left(\frac{r_{g}\left(x\right)}{r_{f}\left(x\right)}-\log\frac{r_{g}\left(x\right)}{r_{f}\left(x\right)}-1\right)dx. \end{array}$$

In a similar context, Lemma 2 and Theorem 1 says that the approximation of the quantal Kullback-Leibler information can also be represented in terms of the hazard function and reverse hazard function; hence, we can expect the following quantal KL information representation in terms of the hazard function and reverse hazard function.

**Theorem** **2.** (13) $$\begin{array}{ccc} QKL(F:G) & = & \int_{-\infty }^{\infty }W\left(x\right)f\left(x\right)\left\{\frac{r_{g}\left(x\right)}{r_{f}\left(x\right)}-\log\frac{r_{g}\left(x\right)}{r_{f}\left(x\right)}-1\right\}dx\\ & & -\int_{-\infty }^{\infty }W\left(x\right)f\left(x\right)\left\{\frac{h_{g}\left(x\right)}{h_{f}\left(x\right)}-\log\frac{h_{g}\left(x\right)}{h_{f}\left(x\right)}-1\right\}dx. \end{array}$$

**Proof** **of** **Theorem 2.** We can show that

$$\begin{array}{ccc} \frac{d}{dx}\left\{F\left(x\right)\log\frac{F(x)}{G(x)}\right\} & = & -f\left(x\right)\left\{\frac{r_{g}\left(x\right)}{r_{f}\left(x\right)}-\log\frac{r_{g}\left(x\right)}{r_{f}\left(x\right)}-1+\log\frac{g(x)}{f(x)}\right\}\\ \frac{d}{dx}\left\{\bar{F}\left(x\right)\log\frac{\bar{F}\left(x\right)}{\bar{G}\left(x\right)}\right\} & = & f\left(x\right)\left\{\frac{h_{g}\left(x\right)}{h_{f}\left(x\right)}-\log\frac{h_{g}\left(x\right)}{h_{f}\left(x\right)}-1+\log\frac{g(x)}{f(x)}\right\}. \end{array}$$

Then, we can apply the integration by parts to (2) to get the result. □

Equation (13) can be rewritten in terms of the cumulative distribution function as follows:

$$\begin{array}{c} QKL\left(F:G\right)=\int_{-\infty }^{\infty }W\left(x\right)\frac{F(x)-G(x)}{G(x)(1-G(x))}dG\left(x\right)+\int_{-\infty }^{\infty }W\left(x\right)\log\frac{G(x)/(1-G(x))}{F(x)/(1-F(x))}dF\left(x\right). \end{array}$$

Hence, the quantal KL information has another representation in terms of the cumulative distribution function, which measures the weighted differences in distribution functions and the log odds ratio.

Now, we consider the choice of the weight function W(x) in QKL(F:G), which has not been discussed much so far. Here, we consider the criterion of maximizing the quantal Fisher information in Theorem 1. For the multi-parameter case, we have the quantal Fisher information matrix and can consider its determinant, which is called *generalized Fisher information*.

**Figure 1:**
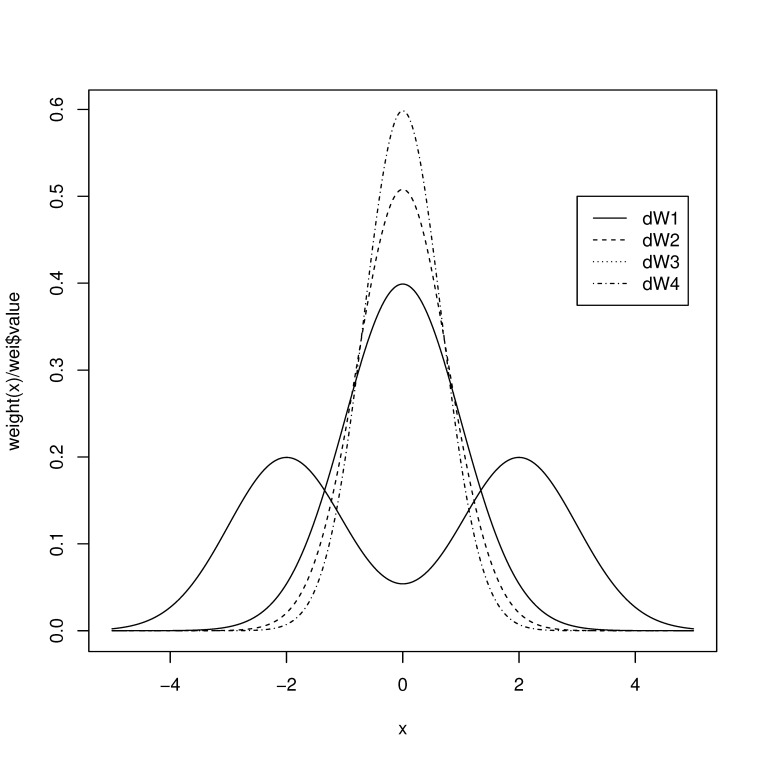
Shapes of the chosen weight functions.

For illustration, we take F(x) to be the normal distribution. Then we consider the following dW(x)’s and plotted their shapes in Figure 1 where $dW_{1}\left(x\right)$ is the bimodal weight function and the shapes get more centralized as *i* in $dW_{i}\left(x\right)$ increases.

1. $dW_{1}\left(x\right)=d\left\{0.5\Phi \left(x-2\right)+0.5\Phi \left(x+2\right)\right\}$
2. $dW_{2}\left(x\right)=\pi /8\times \Phi {\left(x\right)}^{0.5}{\left(1-\Phi \left(x\right)\right)}^{0.5}d\Phi \left(x\right)$
3. $dW_{3}\left(x\right)=d\Phi \left(x\right)$
4. $dW_{4}\left(x\right)=6\Phi \left(x\right)\left(1-\Phi \left(x\right)\right)d\Phi \left(x\right)$,

where Φ(x) is the cumulative distribution function of the normal random variable.

We calculate the corresponding quantal Fisher information and summarize the results in Table 1. We can see from Table 1 that $I_{QF}\left(\theta \right)$ about the location parameter gets larger as the weight function becomes more centralized. We also note that we have the maximum $I_{QF}\left(\theta \right)$ about the scale parameter at the bimodal weight function. However, we can see that we have the maximum generalized quantal Fisher information at dW(x)=dΦ(x).

## 5. Estimation of the Quantal KL Information

Suppose that we have an independently and identically distributed (IID) sample of size *n*, $\left(x_{1},\cdots ,x_{n}\right)$, from an assumed density function $f_{\theta }\left(x\right)$, and $\left(x_{1:n},\cdots ,x_{n:n}\right)$ are their ordered values. Then, the distance between the sample distribution and the assumed distribution can be measured as $KL(f_{n}:f_{\theta })$, where $f_{n}$ is an appropriate nonparametric density function estimator, and its estimate has been studied as a goodness-of-fit test statistic by lots of authors, including Pakyari and Balakrishnan [13], Noughabi and Arghami [14], and Qiu and Jia [15] by considering a piecewise uniform density function estimator or nonparametric kernel density function estimator. In the same manner, the estimate of (12) has been studied by Park and Shin (2015) for the same purpose by considering a nonparametric hazard function estimator. However, we note that the critical values based on those nonparametric density (hazard) function estimators depend on the choice of the bandwidth-type parameter.

We can also measure the distance between the sample distribution and the assumed distribution with $QKL(F_{n}:F_{\theta })$, if we choose the weight function to be $F_{n}\left(x\right)$ in view of Section 4, which can be written as

(14) $$\begin{array}{c} QKL\left(F_{n}:F_{\theta }\right)=\int_{-\infty }^{\infty }\left(F_{n}\left(x\right)\log\frac{F_{n}\left(x\right)}{F_{\theta }\left(x\right)}+{\bar{F}}_{n}\left(x\right)\log\frac{{\bar{F}}_{n}\left(x\right)}{\bar{F_{\theta }}\left(x\right)}\right)dF_{n}\left(x\right), \end{array}$$

where $F_{n}$ is the empirical distribution function.

Then, ${\bar{F}}_{n}\left(x_{i:n}\right)$ can be obtained as i/n, and $dF_{n}\left(x\right)$ is obtained as 1/n only at $x_{i:n}$’s, and (14) can be written as

$$\begin{array}{ccc} QKL_{R}\left(F_{n}:F_{\theta }\right) & = & \sum_{i=1}^{n}\left\{\frac{i}{n}\log\frac{i/n}{\xi_{i}}+\frac{n-i}{n}\log\frac{(n-i)/n}{1-\xi_{i}}\right\}\\ & = & -\frac{1}{n}\sum_{i=1}^{n}\left\{i\log\xi_{i}+\left(n-i\right)\log\left(1-\xi_{i}\right)\right\}+C_{1}, \end{array}$$

where $\xi_{i}=F_{\theta }\left(x_{i:n}\right)$, $\xi_{0}=0$ and $\xi_{n+1}=1$, and $C_{1}=\sum_{i=1}^{n}\left\{\left(i/n\right)\log\left(i/n\right)+\left(1-i/n\right)\log\left(1-i/n\right)\right\}$.

However, because the empirical distribution function is only right-continuous, we also consider ${\bar{F}}_{n}\left(x_{i:n}\right)$ to be (n−i+1)/n so that ${\bar{F}}_{n}\left(x_{i:n}\right)$ to be (i−1)/n, then we have

$$\begin{array}{ccc} QKL_{L}\left(F_{n}:F_{\theta }\right) & = & \sum_{i=1}^{n}\left\{\frac{i-1}{n}\log\frac{(i-1)/n}{\xi_{i}}+\frac{n-i+1}{n}\log\frac{(n-i+1)/n}{1-\xi_{i}}\right\}\\ & = & -\frac{1}{n}\sum_{i=1}^{n}\left\{\left(i-1\right)\log\xi_{i}+\left(n-i+1\right)\log\left(1-\xi_{i}\right)\right\}+C_{1}. \end{array}$$

Hence, we may obtain the average of both and obtain

$$\begin{array}{c} QKL_{n}\left(F_{n}:F_{\theta }\right)=-\frac{1}{n}\sum_{i=1}^{n}\left\{\left(i-0.5\right)\log\xi_{i}+\left(n-i+0.5\right)\log\left(1-\xi_{i}\right)\right\}+C_{1}, \end{array}$$

which is actually equivalent to the Anderson-Darling test.

Zhang [10] proposed a test statistic by choosing a weight function,

$$\begin{array}{c} dW\left(x\right)=1/\left\{F_{n}\left(x\right){\bar{F}}_{n}\left(x\right)\right\}dF_{n}\left(x\right), \end{array}$$

as

$$\begin{array}{c} Z_{A}=-\sum_{i=1}^{n}\left\{\frac{\log\xi_{i}}{n-i+0.5}+\frac{\log(1-\xi_{i})}{i-0.5}\right\}+C_{2}, \end{array}$$

where $C_{2}=\sum_{i=1}^{n}\left\{\log\left(\left(i-0.5\right)/n\right)/\left(\left(n-i+0.5\right)/n\right)+\log\left(\left(n-i+0.5\right)/n\right)/\left(\left(i-0.5\right)/n\right)\right\}$.

For example, we consider the performance of the above statistics for testing the following hypothesis:

$H_{0}$: The true distribution function is N(μ,σ)

versus

$H_{1}$: The true distribution function is not N(μ,σ).

The unknown parameters, μ and σ, are estimated with the sample mean and sample standard deviation, respectively. We also consider the classical Kolmogorov-Smirnov test statistic (Lilliefors test) for comparison as

$$\begin{array}{c} KS=\underset{z}{\sup}\left|F_{n}\left(z\right)-N\left(0,1\right)\right|, \end{array}$$

where $z=(x-\bar{x})/s$ and $\bar{x}$ and *s* are the sample mean and the sample standard deviation, respectively.

We provide the critical values of the above test statistics for n=10,20,⋯,100 in Table 2, which are obtained by employing the Monte Carlo simulations of size 200,000.

Then, we compare the power estimates of the above test statistics, for illustration, against the following alternatives to compare the powers:

1. Symmetric alternatives: Logistic, t(5),t(3),t(1), Uniform, Beta(0.5,0.5), Beta(2,2);
2. Asymmetric alternatives: Beta(2,5), Beta(5,2), Exponential, Lognormal(0,0.5), Lognormal(0,1).

We also employed the Monte Carlo simulation to estimate the powers against the above alternatives for n=20,50,100, respectively, where the simiulation size is 100,000. The numerical results are summarized in Table 3, Table 4 and Table 5. These show that $QKL_{n}$ performs better than $QKL_{R}$ and $QKL_{L}$ against symmetric alternatives, and the powers of $QKL_{n}$ against asymmetric alternatives are in between $QKL_{R}$ and $QKL_{L}$. They all outperform the classical Kolmogorov-Smirnov test statistic. $Z_{A}$ generally performs better than $QKL_{n}$ against asymmetric alternatives, but the simulation result shows that $Z_{A}$ seems to be a biased test, which can be known from the power estimate against Beta(2,2) for n=20.

## 6. Concluding Remarks

It is well-known that both Fisher information and Kullback-Leibler information can be in terms of the hazard function or reverse hazard function. We considered the quantal response variable and showed that the quantal Fisher information and quantal KL information can also be represented in terms of both hazard function and reverse hazard function. We also provided the criterion of maximizing the standardized quantal Fisher information in choosing the weight function in the quantal KL information. For illustration, we considered the normal distribution and studied the choice of weight function, and compared the performance of the estimators of the quantal KL information as a goodness-of-fit test.

## Figures and Tables

**Table 1:** Quantal FI for some weight functions.

Specification		$I_{\mathrm{QF}}\left(\theta \right)$			
**Distribution**	**Parameter**	${\mathrm{dW}}_{1}\left(x\right)$	${\mathrm{dW}}_{2}\left(x\right)$	${\mathrm{dW}}_{3}\left(x\right)$	${\mathrm{dW}}_{4}\left(x\right)$
Normal	Location	0.1983	0.4205	0.4805	0.5513
Normal	Scale	0.3497	0.3014	0.2701	0.1883
Normal	Generalized FI	0.0693	0.1267	0.1298	0.1013

**Table 2:** Critical values of test statistics.

*n*	${\mathrm{QKL}}_{R}$	${\mathrm{QKL}}_{L}$	${\mathrm{QKL}}_{n}\left({\mathrm{AD}}_{n}\right)$	$Z_{A}$	KS
10	0.4360	0.4350	0.4322	2.7395	0.2619
20	0.4131	0.4141	0.4115	3.9108	0.1920
30	0.4028	0.4025	0.4008	4.6310	0.1586
40	0.3977	0.3978	0.3960	5.1486	0.1385
50	0.3928	0.3924	0.3918	5.5000	0.1244
60	0.3901	0.3905	0.3892	5.8764	0.1138
70	0.3914	0.3914	0.3905	6.1723	0.1060
80	0.3872	0.3873	0.3867	6.3988	0.0991
90	0.3874	0.3874	0.3867	6.6307	0.0935
100	0.3858	0.3851	0.3851	6.8319	0.0888

**Table 3:** Power estimate (%) of 0.05 tests against 10 alternatives of the normal distribution based on 100,000 simulations; n=20.

Alternatives	${\mathrm{QKL}}_{R}$	${\mathrm{QKL}}_{L}$	${\mathrm{QKL}}_{n}\left({\mathrm{AD}}_{n}\right)$	$Z_{A}$	KS
N(0,1)	5.01	5.01	5.00	5.08	4.96
Logistic(0,1)	10.54	10.35	10.48	12.34	8.55
t(5)	16.98	16.86	17.04	19.69	13.15
t(3)	32.15	31.96	32.35	34.56	26.03
t(1)	88.06	88.04	88.23	86.49	84.63
Uniform	16.57	16.40	16.78	13.57	9.71
Beta(0.5,0.5)	60.66	60.52	61.10	66.55	31.82
Beta(1,1)	16.73	16.55	16.91	13.53	9.89
Beta(2,2)	5.52	5.41	5.52	3.26	5.08
Beta(2,5)	11.53	17.47	14.64	17.26	11.54
Beta(5,2)	17.92	11.48	14.77	17.62	11.51
Exponential(1)	72.62	81.23	77.59	86.72	58.54
Log normal(0,0.5)	40.64	51.40	46.64	53.98	34.29
Log normal(0,1)	88.03	92.20	90.48	94.32	79.20

**Table 4:** Power estimate (%) of 0.05 tests against 10 alternatives of the normal distribution based on 100,000 simulations; n=50.

Alternatives	${\mathrm{QKL}}_{R}$	${\mathrm{QKL}}_{L}$	${\mathrm{QKL}}_{n}\left({\mathrm{AD}}_{n}\right)$	$Z_{A}$	KS
N(0,1)	5.06	5.12	5.05	5.16	5.05
Logistic(0,1)	16.13	16.21	16.20	18.49	11.45
t(5)	30.25	30.31	30.41	33.63	21.10
t(3)	60.86	60.85	60.99	61.60	48.57
t(1)	99.72	99.72	99.73	99.48	99.33
Uniform	57.43	57.61	57.73	80.08	25.92
Beta(0.5,0.5)	99.06	99.08	99.08	99.97	80.21
Beta(1,1)	57.54	57.59	57.80	80.04	26.07
Beta(2,2)	13.18	13.30	13.33	14.75	8.21
Beta(2,5)	35.09	43.79	39.67	59.41	25.65
Beta(5,2)	43.56	35.09	39.47	59.03	25.57
Exponential(1)	99.50	99.76	99.65	99.99	96.05
Log normal(0,0.5)	84.70	89.52	87.40	94.16	71.05
Log normal(0,1)	99.94	99.97	99.96	100.00	99.52

**Table 5:** Power estimate (%) of 0.05 tests against 10 alternatives of the normal distribution based on 100,000 simulations; n=100.

Alternatives	${\mathrm{QKL}}_{R}$	${\mathrm{QKL}}_{L}$	${\mathrm{QKL}}_{n}\left({\mathrm{AD}}_{n}\right)$	$Z_{A}$	KS
N(0,1)	5.06	5.08	5.04	5.05	5.05
Logistic(0,1)	24.15	24.16	24.19	24.99	15.57
t(5)	48.18	48.23	48.26	50.34	33.23
t(3)	84.94	84.97	84.97	83.52	73.09
t(1)	100.00	100.00	100.00	100.00	100.00
Uniform	95.02	95.07	95.07	99.93	59.20
Beta(0.5,0.5)	100.00	100.00	100.00	100.00	99.47
Beta(1,1)	94.82	94.89	94.88	99.95	58.92
Beta(2,2)	31.96	32.10	32.10	54.81	15.39
Beta(2,5)	72.88	78.80	76.00	96.22	50.56
Beta(5,2)	78.73	72.98	76.04	96.11	50.70
Exponential(1)	100.00	100.00	100.00	100.00	100.00
Log normal(0,0.5)	99.28	99.59	99.47	99.94	95.07
Log normal(0,1)	100.00	100.00	100.00	100.00	100.00

## Data Availability

Not applicable.
